# Assessment of the Perception of People Living With HIV Regarding the Quality of Outpatient Care at a Reference Facility in the Federal District, Brazil

**DOI:** 10.3389/fphar.2021.740383

**Published:** 2021-09-20

**Authors:** Andressa Wanneska Martins da Silva, Micheline Marie Milward de Azevedo Meiners, Luciana Guerra Gallo, Ana Flávia de Morais Oliveira, Ursila Manga Aridja, Elza Ferreira Noronha

**Affiliations:** Tropical Medicine, Faculty of Medicine, University of Brasília, Brasília, Brazil

**Keywords:** HIV, outpatient care, healthcare models, assessment of caregiver quality, patient assessment of chronic illness care

## Abstract

The effectiveness of antiretroviral treatment has transformed HIV infection into a chronic transmissible condition, requiring health systems to adapt in order to care for people living with HIV. The Chronic Care Model (CCM) is the gold standard for this type of care in many countries. Among its tools, the Patient Assessment of Chronic Illness Care (PACIC) questionnaire gives the patient’s perspective of the care provided. The aim of the present study was to adapt and apply, for the first time, the questionnaire to people living with HIV to determine their perception of the quality of care provided at a reference hospital in the Federal District of Brazil. This is a case study conducted in 2019 at a teaching hospital, with a convenience sample of 30 individuals treated for at least 1 year at the facility. The median PACIC score (3.5 with a range of 1.0–5.0) seems to suggest that the users perceive the outpatient care provided by the hospital as being basic. The “delivery system design/decision support” component was deemed the best (5.0, with a range of 1.0–5.0) and “follow-up/coordination” the worst (1.0, with a range of 1.0–5.0). The results suggest the need to improve the organization of care and make adequate use of community resources, in line with the CCM. The questionnaire makes it possible to determine the strengths and weaknesses of the care provided to people living with HIV and can be used as a planning and monitoring tool to improve management of the condition, with the contribution of the patient, in particular, thereby strengthening self-care.

## Introduction

Since the onset of the epidemic, HIV has caused around 32 million deaths worldwide (World Health Organization, 2019). Estimates of the prevalence of people living with HIV (PLHIV) worldwide and in Brazil were 0.49 and 0.41%, respectively at the end of 2018 (World Health Organization, 2019).

Due to the effectiveness of preventive measures, diagnoses and health care in PLHIV, the condition is now considered chronic, that is, involving continuous management for several years (Schaurich et al., 2006; Yehia et al., 2008; Swendeman et al., 2009; Rabkin and Nishtar, 2011; Deeks et al., 2014).

One of the tools of the Chronic Care Model (CCM), used internationally to organize chronic care, is the Patient Assessment of Chronic Illness Care (PACIC) (Wagner, 1998; Wagner et al., 2001; Bodenheimer et al., 2002). The PACIC has previously been applied for a number of chronic nontransmissible conditions (Aragones et al., 2008; Schmittdiel et al., 2008; Steurer-Stey et al., 2012; Abbott et al., 2018; Desmedt et al., 2018; Pilipovic-Broceta et al., 2018). In Brazil, PACIC has been applied mainly to the diseases diabetes and hypertension (Moysés et al., 2012; Castro et al., 2018; Silva et al., 2018). The questionnaire evaluates the quality of care from the patient’s perspective, assessing the components of the CCM and the performance of teams in promoting self-care (Wagner et al., 2001; Glasgow et al., 2005).

However, to date, no studies have applied the PACIC to PLHIV. Using the PACIC in this condition could determine patient perception and help health facilities monitor the quality of care and support self-care. The involvement of PLHIV in self-care, respecting and encouraging their autonomy as co-responsible in the treatment, is important in ensuring they obtain better clinical outcomes (Wagner et al., 1996; Glasgow et al., 2006).

In 2000, the Ministry of Health, in partnership with research groups from public Brazilian universities, developed Qualiaids, a questionnaire to assess the quality of outpatient care for PLHIV. However, this instrument does not take the patient’s perspective into account (Qualiaids, 2019). Thus, the PACIC could be considered a complement to Qualiaids in assessing HIV outpatient services. In addition to evaluating care quality, the PACIC can guide interventions into the interpersonal aspects that influence adequate management of the condition (Wagner et al., 1996; Wagner, 1998; Glasgow et al., 2005; Tu et al., 2013; Abdulrahman et al., 2019). These aspects are not addressed in the Brazilian Qualiaids questionnaire.

The following criteria were considered to compare the present study with others in the literature: having applied the PACIC to any chronic illness and being a Brazilian study. According to the results of a systematic review with meta-analysis, the PACIC was not created for comparisons between countries, since how cultural and idiomatic factors affect the overall score remains unknown (Arditi et al., 2018). A comprehensive review on PACIC application showed that the questionnaire is little used in the world. Between 2005 and 2018, a mere 85 studies were found, only two of which were Brazilian (Arantes et al., 2019; Schwab et al., 2014; Silva et al., 2018). None of the studies were conducted in PLHIV.

Thus, the aim of the present study was to adapt and apply the PACIC to PLHIV, for the first time, in order to determine their perception of the quality of outpatient care provided at a reference hospital in the Federal District.

## Materials and Methods

### Study Design, Location and Population

This is a descriptive case study, conducted between April and May 2019, at University of Brasilia Hospital (UBH), a reference hospital for the care of people living with HIV in the Federal District and managed by the Brazilian Company for Hospital Services (Ebserh/MEC) since 2013, employs over 3,000 professionals with different affiliations (Ebserh, University of Brasilia, Ministry of Health and outsourced companies), in addition to professors, residents and interns, providing outpatient treatment and hospitalization for medium and high complexity conditions across a wide range of specialties (Brasil, 2020). Outpatient treatment is provided at the institution, involving three healthcare locations: the infectology outpatient clinic, antiretroviral (ART) dispensing unit (Pharmacy) and psychosocial treatment center.

**FIGURE 1:**
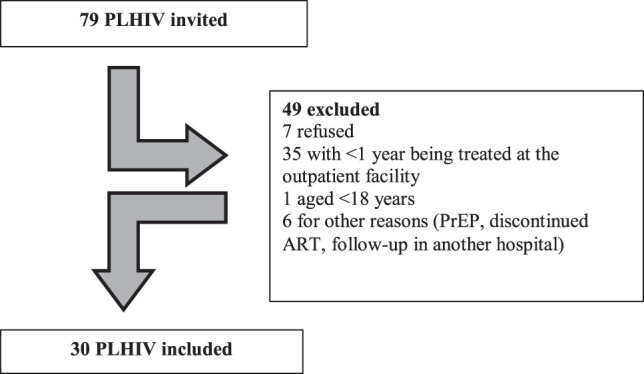
Sample composition of people living with HIV who completed the Patient Assessment of Chronic Illness Care (PACIC), Brazil, 2019. Legend: PLHIV—people living with HIV; PrEP—pre-exposure prophylaxis; ART—antiretroviral therapy.

A total of 79 people were recruited on 10 non-consecutive days over a 2-months period, according to the medical schedule or dispensing routine. Of these, 30 PLHIV met the inclusion criteria and participated as a convenience sample for this study (Figure 1). As such, the sample was only representative of the reality of this particular hospital. There was no set saturation point and the field approaches were not established solely in terms of numbers. The PACIC is a qualitative tool that has been coded to aid in interpretation and comparison with other studies.

The following inclusion criteria were established by the researchers: being diagnosed with HIV, aged 18 years or older, enrolled at the infectology outpatient clinic for at least 1 year before data collection and having had at least one visit with an infectologist during this period. Pregnant women and PLHIV who abandoned treatment were excluded.

It was assumed that the infectologists’ recommendations (every 6 months) and ART dispensing (every 2 months) may promote sufficient contacts with the outpatient facility, that is, bond and regular follow-up, so that PLHIV can express their perception (Brasil, 2018). On the other hand, abandonment was considered when the PLHIV did not visit the facility for 3 months after interrupting their medication or stayed away for more than 6 months (Brasil, 2009).

### Sample Recruitment and Selection

The volunteers were recruited at the infectology outpatient clinic and the Pharmacy. At the outpatient facility, collection occurred by examining the list of scheduled doctors’ appointments, determining whether patients met the inclusion criteria, to be subsequently invited to participate in the study and undergo the PACIC. At the Pharmacy, collection occurred with the support of the health team, which indicated likely candidates to the researchers. These candidates were approached directly and asked the following questions – “Are you treated at the infectology outpatient clinic?.” If so, “since when?.” If eligible, those who agreed to participate completed the PACIC. Next, the medical charts of the participants were examined to determine if they met the inclusion criteria.

### Information Sources

The University Hospital Management App (AGHU) was used to obtain sociodemographic (sex, age, skin color, place of residence) and clinical variables (mental health, viral load, regular follow-up, comorbidities, adverse reactions to medication, antiretroviral scheme, time since diagnosis) from the online medical charts.

For other pertinent information, the researchers created a set of questions to determine any access to other specialties and examinations, length of time being treated at the hospital, number of psychosocial visits, number of visits to the Pharmacy and number of laboratory examinations in the previous year. These questions were asked before the PACIC was applied. For more information about the survey, see Supplementary Material S1.

### Patient Assessment of Chronic Illness Care

The PACIC aims at determining the patients’ perception of the quality of care provided by the health team and their involvement in supporting self-care. It is one of the assessment tools devised for the Chronic Care Model (CCM) developed by the research team from the MacColl Center for Health Care Innovation (Glasgow et al., 2005). In regard to reliability, the original PACIC instrument showed internal consistency of 0.93 based on Cronbach’s alpha and 0.84 for the tool translated into Brazilian Portuguese for people with diabetes (Glasgow et al., 2005; Castro et al., 2018). The Portuguese version of the questionnaire was translated, adapted and validated for use with patients enrolled in the Brazilian national health system (SUS) (Moysés et al., 2012). The Brazilian version of the PACIC was deemed adequate for application in PLHIV, since it followed WHO translation guidelines and considered the profile of the population vulnerable to chronic conditions who use the health services in the country (Moysés et al., 2012).

In the present study, the Portuguese version of the questionnaire was adapted and tested for people living with HIV (Supplementary Appendix S1). Adaptations consisted of replacing a few words, such as the term “chronic illness” with “HIV”, and “health problems” with “my health”; and a list of support groups for the self-care of HIV was also compiled, to facilitate understanding and interpreting the questions.

After the adaptations, we conducted a comprehension test of the PACIC questionnaire in two rounds of seven and nine participants, respectively, using the same inclusion criteria applied in the study. The participants were recruited during antiretroviral dispensing at the Pharmacy.

In the first round, each question was read and the volunteers were asked if they understood and knew how to answer. If not, they were asked what they did not understand. The questions were well understood by most participants. Five PLHIV in the first round did not understand one question each (1, 6, 7, 12, and 13), out of a total of 20 questions.

Thus, minor changes were made to the questionnaire for the second round, for example: from “my ideas” to “my opinion” in item one, adding “for HIV” to some phrases to characterize the condition and changing “my condition” to “my health”. Since no misunderstandings were pointed out in the second round, the questionnaire was considered suitable for application.

The questionnaire contains five analysis categories/components and 20 questions, according to the theoretical framework of the Chronic Care Model (CCM) (Glasgow et al., 2005), as follows: 1) patient activation (assesses how much motivation and professional support the individual received to initiate changes), with three questions; 2) delivery system design/decision support (assesses the support the individual received for their care, such as educational materials, invitation to the roundtable discussion, and how satisfied they were with the organization of the care provided), with three questions; 3) goal setting (assesses the extent to which general instructions, clinical protocols and suggestions were adapted to the patient’s situation), with five questions; 4) problem solving/contextual counseling (assesses how the health team deals with the problems that compromise achieving the established goals), with four questions; and 5) follow-up/coordination (assesses the frequency and consistency of the care), with five questions. For each question, the answers vary from 1 to 5 (Likert scale: none of the time, a little of the time, some of the time, most of the time, always) (Glasgow et al., 2005).

Before the PACIC was applied, it was made clear to the respondents that they could ask the researcher if they were unsure about a question. Next, each question was read aloud and the respondent selected the answer that best described their experience with the outpatient care among one to 5. The PACIC took an average of 15 min to complete.

### Data Analysis

The PACIC questionnaire data and those collected from the medical charts and set of questions were tabulated and analyzed using the Statistical Package for the Social Sciences® (SPSS), version 22.0 (IBM Corp, NY, United States) applying descriptive statistics. For the sociodemographic, clinical and access-to-services variables, the median and range of variation (minimum and maximum value or quartiles) and percentages were used. The following was obtained for the PACIC: 1) for each question, the percentage of answers for each Likert category; 2) for each of the components, the median, minimum and maximum scores; and 3) for each questionnaire, the median, minimum and maximum scores. According to the authors, the highest scores indicate better patient perception of team involvement in the self-care and support for their condition (Glasgow et al., 2005). Care quality was classified according to the median score obtained, as follows: limited quality = 1.0–2.9; basic quality = 3.0–3.9 and good/excellent quality = 4.0–5.0.

## Results

During the study, 79 PLHIV were invited to take part, but only 30 were included, as shown in Figure 1.

Based on the results presented in Table 1, the median age of the subjects was 42.5 years (q1-q3, 29.5–48.3), with greater frequency of men and schooling level not contained on the medical charts, non-white skin color, and residents in the Southwest region. The medians for time since diagnosis and time enrolled at the outpatient clinic were 4.0 (2.3–13.3) and 4.5 years (2.0–10.0), respectively. Only three people reported an adverse reaction to ART. According to the medical charts, more than 76% exhibited undetectable viral load, even though around 35% used the preferential scheme in line with the Brazilian protocol (Brasil, 2018). Around 75% of the PLHIV had regular follow-up to ART and several exhibited comorbidities (63.3%).

**TABLE 1:** Sociodemographic and clinical characteristics and access to outpatient care of people living with HIV (*n* = 30), Brazil, 2019.

Age - median (q1-q3)		42.5 (29.5–48.3)
		**Frequency (%)**
Sex	Male	25 (83.3)
Schooling	Unknown	22 (73.3)
Race/color	Non-white	20 (66.7)
Place of residence^a^	Southeast	6 (20.0)
	East	5 (16.6)
	Center	5 (16.6)
Regular follow-up^b^	Yes	23 (76.7)
Viral load	Undetectable	23 (76.7)
Antiretroviral therapy^c^	Preferential^d^	11 (36.7)
Adverse reaction to ART	Yes	3 (10.0)
Mental health^e^	Unchanged	28 (93.3)
Comorbidity	Yes	19 (63.3)
Access to other specialties^f^	Obtained	16 (53.3)
		**Median (q1-q3)**
Time since diagnosis in years		4.0 (2.3–13.3)
Time under treatment at the hospital in years		4.5 (2.0–10.0)
Number of infectologist visits^f^		3.0 (2.0.-3.0)
Number of psychosocial visits^f^		0.0 (0.0–2.0)
Number of visits to the Pharmacy^f^		12.0 (7.0–12.0)
Number of laboratory visits^f^		2.0 (1.0–3.0)

a Health Regions: Southeast (Taguatinga, Águas Claras, Vicente Pires and Samambaia), West (Ceilândia and Brazlândia), East (Paranoá, Itapoã, São Sebastião, Lago Sul and Jardim Botânico), South (Gama and Santa Maria), North (Planaltinha, Sobradinho and Fercal), Center-South (Candangolândia, Estrutural, Guará, Park Way, Núcleo Bandeirante, Riacho Fundo I and II, Setor de Indústria and Abastecimento (SIA) and Setor Complementar de Indústria and Abastecimento (SCIA) and Center (Northwest, Asa Sul and Asa Norte) (Brasil, 2019a; Brasil, 2019b).

b Regular follow-up: patients under regular treatment at the Pharmacy and undetectable viral load.

c ART, Antiretroviral therapy.

d Preferential: tenofovir + lamivudine and dolutegravir.

e For mental health, CID 10: F41-other anxious disorders were considered.

f Data collected from the last year of follow-up.

With respect to access, slightly more than half reported having access to other specialties or examinations in the hospital. There were a median of three infectologist visits per year, monthly dispensing of medication, but few or no psychosocial visits. It is important to underscore that more than 90% of the sample had no registered mental problems.

**FIGURE 2:**
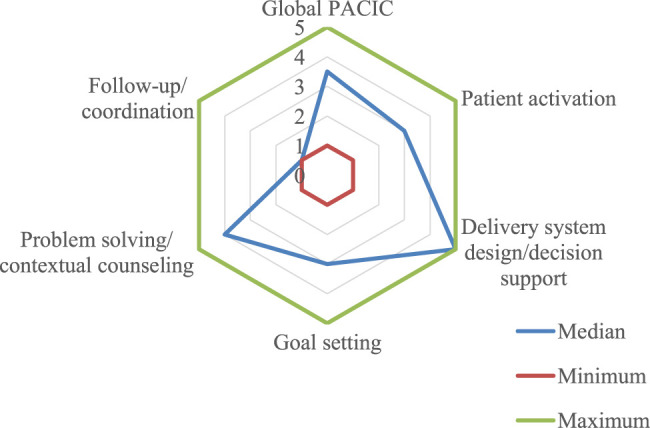
Overall PACIC score^1,2,3^ per component (*n* = 30) Brazil, 2019. Legend: ^1^ PACIC - Patient Assessment of Chronic Illness Care. ^2^ PACIC response categories: 1-none of the time, 2-a little of the time, 3-some of the time, 4-most of the time, 5-always. ^3^ Answer categories created by the authors: 1.0–2.9 limited quality, 3.0–3.9 basic quality, 4.0–5.0 excellent quality.

**FIGURE 3:**
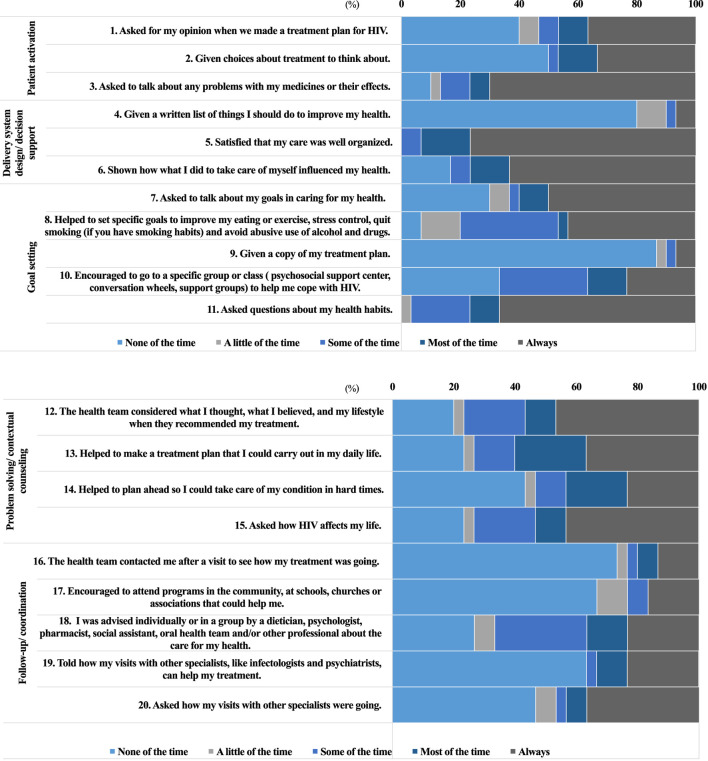
Descriptive distribution of PACIC^1,2^ per question according to the five components (*n* = 30), Brazil, 2019.^1^ PACIC: Patient Assessment of Chronic Illness Care. ^2^ PACIC response categories: 1-none of the time, 2-a little of the time, 3-some of the time, 4-most of the time, 5-always (continue).

The median PACIC score (3.5; min-max: 1.0–5.0) showed that the patient perceived the quality of outpatient care provided by the hospital as basic (Figure 2). The median of each component was lowest for “follow-up/coordination” and highest for “delivery system design/decision support” (Figure 3).

Considering each of the component questions, with respect to “patient activation”, more than 75% of PLHIV reported that they were asked by the health team about any problems in the use of medication or their effects “most of the time” or “always”. On the other hand, nearly 50% of patients answered that they had the right to information on the therapeutic plan “none of the time” or “a little of the time” (questions 1 and 2) (Figure 3). This component obtained a median of 3.0 (min-max: 1.0–5.0) and was therefore assessed as exhibiting basic quality (Figure 2).

The “delivery system design/decision support” component (5.0; min-max: 1.0–5.0) obtained one of the highest scores. More than 75% of respondents were satisfied with the organization of treatment and received information on how self-care could influence HIV management. However, this information was not usually provided in written form (Figure 3).

The non-availability of written information was also observed in the “goal setting” component, where 87% of PLHIV reported not receiving the treatment plan. It was also noted that the health team discussed health habits with the patients (Figure 3). This component obtained a median of 3.0 (min-max: 1.0–5.0) (Figure 2).

Nearly 50% of patients reported that they “always” felt supported by the health team in solving problems related to HIV management, except during what they considered “hard times” (43%) (Figure 3). The “problem solving/contextual counseling” component obtained a median of 4.0 (min-max: 1.0–5.0) and lowest variability in the group (Figure 2).

The worst assessed component, with limited quality, was “follow-up/coordination” which obtained a median of 1.0 (min-max: 1.0–5.0) (Figure 2). Although most of the PLHIV received multiprofessional care, many were not contacted by the team to assess treatment evolution after a visit (73%) and did not feel encouraged to attend community programs (67%) (Figure 3).

## Discussion

The PACIC results showed that the people living with HIV treated at this reference hospital considered that the quality of the outpatient care provided was basic. The “delivery system design/decision support” component received the best assessment, and “follow-up/coordination” the worst.

Based on the PACIC results, patients considered the quality of the outpatient care provided to PLHIV to be basic (3.5; min-max: 1.0–5.0) that is, there is still room to improve the healthcare measures implemented, particularly those not identified by users of the service. A number of Brazilian studies that applied the PACIC to National Health System (SUS) patients under treatment for chronic conditions, primarily people with diabetes and hypertension, obtained results that ranged from limited to basic quality, similar to our findings (Moysés et al., 2012; Castro et al., 2018; Silva et al., 2018). In terms of public health, the quality of the services provided by the hospital, as perceived by PLHIV, is comparable to that offered by the SUS for other chronic illnesses.

Analysis of each component of the questionnaire shows that in “patient activation”, most PLHIV reported that they were asked by the health team about their problems in using drugs or their side effects “most of the time” or “always”. This is confirmed by the clinical findings for regular follow-up, undetectable viral load and alternative treatment in most of the participants. Most of the patients under alternative ART indicated the need to change medication because of adverse reactions or therapeutic failure (salvage therapy) (Brasil, 2018). In a study conducted with diabetic patients, most of the participants indicated that the professionals “always” asked them about possible problems with the medication (Castro et al., 2018). However, in another study carried out with diabetic and hypertensive patients, most reported that this question is never a priority during treatment (Silva et al., 2018). Adverse reactions are one of the causes of non-adherence to HIV treatment (Silva et al., 2015); as such, it is assumed that guaranteeing regular follow-up should be one of the priorities of the multidisciplinary team. When changing medication, the new therapeutic plan should be reinforced with the participation of the entire multiprofessional team, especially pharmacists, in order to help overcome insecurities about initiating a new therapy (fear of new adverse reactions) and uncertainties regarding the new scheme, in addition to reinforcing the importance of treatment adherence (Brasil, 2007).

On the other hand, nearly half of the patients reported that they were only able asked their opinion about their treatment or therapeutic options to establish their therapeutic plan “a little” or “none of the time” (questions 1 and 2). This finding corroborates studies where patient autonomy was assessed as being limited (Moysés et al., 2012; Castro et al., 2018; Silva et al., 2018). It is important to underscore that patients are generally unaware of their rights, especially regarding information and their participation in managing their condition (Ribeiro, 2006; Brasil, 2015). The findings demonstrate the need to respect the right of PLHIV to be informed of the existing protocol and why the ART selected is the best for them.

In the “delivery system design/decision support” component, most of the patients were satisfied with the organization of the treatment and the information received on self-care. Other studies showed that these measures are the best assessed in this component of the questionnaire, having received the highest number of “always” answers (Moysés et al., 2012; Castro et al., 2018; Silva et al., 2018). Patient satisfaction is a complex assessment element. This was confirmed in the present study with the findings of median access to other specialties and examinations and high satisfaction with treatment organization. Effective interactions between the health team and patients, where support for self-care is encouraged, characterize adequate quality for the care of chronic illnesses, reaching better patient satisfaction results and their adherence to the service (Wagner et al., 1996).

On the other hand, in the “goal setting” component, when patients were asked if they received the treatment plan in written form, more than 80% answered “none of the time”. These findings are similar to those of other studies that used the PACIC for different chronic illnesses (Melchior et al., 2006; Castro et al., 2018; Silva et al., 2018). The high demand for treatment and the little time available for consultations may force professionals to prioritize only verbal instructions.

In the results of the “problem solving/contextual counseling” component, patients do not feel supported by the health team in the management of HIV during “hard times.” In the present study, there was low demand for psychosocial support to cope with HIV, given that anxiety and depression are frequent emotional changes among PLHIV (Remor, 1999). The similar proportion in the answer categories of this item, primarily when the “none of the time” and “most of the time/always” groups were compared suggests that 1) help in planning self-care during “hard times” occurs only when requested by the patient; or 2) the health team does not recognize when the PLHIV is in a crisis situation and/or do not feel comfortable intervening. Despite the difficulty defining them in the practice of caregiving, support measures for patients are important throughout life, especially during hard times (Caetano and Pagliuca, 2006; Desmedt et al., 2018).

The “follow-up/coordination” component was the worst assessed, with limited quality (score of less than 3). Although patients reported receiving multiprofessional care, the team does not follow up after the visit. In addition, patients do not feel encouraged to seek the resources available in the community. In a study conducted in 2014 at the same hospital, the institution had limited capacity to forge partnerships with community resources to care for PLHIV (Silva et al., 2019). Similar results were found for other chronic conditions (Moysés et al., 2012; Castro et al., 2018; Silva et al., 2018). These findings may indicate the existence of barriers to continuity of care, lack of partnerships with community resources such as patient associations, churches and non-governmental organizations, and the importance of integration with primary care and health promotion initiatives.

It is believed that the PACIC may help monitor quality of care in terms of organizing health and supporting self-care, since it enriches the overall care provided to PLHIV. In addition, it is proposed that it be used as an assessment tool of the quality of outpatient care to complement evaluation by Qualiaids, which does not address the patient perspective and the interpersonal aspect of quality (Wagner et al., 1996; Wagner, 1998; Glasgow et al., 2005; Tu et al., 2013; Abdulrahman et al., 2019).

Despite the small convenience sample, the researchers found that the sample reached the saturation level corresponding to the perception of PLHIV treated at an outpatient facility of a single health service. However, we cannot state that it is representative of Brazilian people living with HIV. In addition, there was a logistic limitation of human and financial resources to apply the questionnaire that precludes a broader study; however, we underscore the unprecedented use of the tool in HIV, previously applied for other chronic illnesses in Brazil and worldwide. Moreover, there were no discrepancies between the data collected in the interviews, on the medical charts and from the PACIC questions. Although the sample was not representative of the Federal District or Brazil, the data can clarify the perception of care in a group and may result in improvements that benefit the entire study population.

Based on the findings of this study, it is recommended that the PACIC be applied to a representative sample of the population from the hospital or the Federal District. It could also be used in multicenter studies in Brazil in order to assess perception of the quality of outpatient care provided to PLHIV in health systems and different patients, such as those at the onset of treatment, with therapeutic or virologic failure, or comorbidities, among others, and compare the results among them. Additionally, studies should be conducted to determine the association between sociodemographic and clinical variables and access to services, and their influence on the perception of PLHIV regarding the quality of the care received.

## Conclusion

Based on the PACIC results, the people living with HIV treated at this reference hospital considered that the quality of the outpatient care provided was basic. The “delivery system design/decision support” component received the best assessment, and follow-up/coordination the worst. It is believed that the questionnaire may complement Qualiaids in assessing the quality of outpatient care, reinforcing the importance of patient participation at all stages of care.

These findings may help understand the strengths and weaknesses of outpatient care provided to PLHIV, where the aim is to plan and establish strategies and measures to manage this condition, supporting the self-care of patients. Since this is the first time PACIC has been applied to PLHIV, further research is needed to confirm the findings, using a larger number of patients and adequate validation of the questionnaire.

## Data Availability

The original contributions presented in the study are included in the article/Supplementary Material, further inquiries can be directed to the corresponding author.
