# Supplementary material for: Assessment of the Perception of People Living With HIV Regarding the Quality of Outpatient Care at a Reference Facility in the Federal District, Brazil
Source: Front Pharmacol. 2021 Sep 20;12:740383. doi: 10.3389/fphar.2021.740383 (PMC8522475; doi:10.3389/fphar.2021.740383)
Supplement: Supplementary file 1 [file DataSheet2.PDF]

AVALIAÇÃO DO USUÁRIO SOBRE O CUIDADO ÀS CONDIÇÕES CRÔNICAS – PACIC

Manter-se saudável pode ser difícil quando se tem uma condição crônica. Nós gostaríamos de saber como você, nessa condição, é atendido pela equipe de saúde. Isso inclui seu médico, enfermeira e outros profissionais da equipe que tratam seus problemas de saúde. Suas respostas serão confidenciais e não serão compartilhadas com profissionais da equipe.

Durante os últimos seis meses, quando recebeu atendimento para seu problema de saúde:

	Nunca	Poucas vezes	Algumas vezes	Muitas vezes	Sempre
1. Perguntaram minha opinião quando definimos o plano para tratamento de meu problema de saúde. (diabete, hipertensão ou depressão)	☐1	☐2	☐3	☐4	☐5
2. Deram opções de tratamento para que eu pudesse pensar sobre elas.	☐1	☐2	☐3	☐4	☐5
3. Perguntaram se tive problemas no uso dos medicamentos ou seus efeitos.	☐1	☐2	☐3	☐4	☐5
4. Recebi, por escrito, uma lista de coisas que poderia fazer para melhorar minha saúde.	☐1	☐2	☐3	☐4	☐5
5. Fiquei satisfeito com a organização de meu tratamento.	☐1	☐2	☐3	☐4	☐5
6. Explicaram que o que eu faço para cuidar de mim mesmo influencia meu problema de saúde (diabete, hipertensão ou depressão).	☐1	☐2	☐3	☐4	☐5
7. Perguntaram o que eu pretendo fazer para cuidar de meu problema de saúde (diabete, hipertensão ou depressão).	☐1	☐2	☐3	☐4	☐5
8. Ajudaram a definir como melhorar minha alimentação, fazer exercícios físicos, controlar estresse, parar de fumar e evitar o uso abusivo de álcool.	☐1	☐2	☐3	☐4	☐5
9. Recebi uma cópia, por escrito, do plano para tratamento de meu problema de saúde (diabete, hipertensão, depressão).	☐1	☐2	☐3	☐4	☐5
10. Incentivaram a participar de grupos específicos (hipertensos, diabéticos, caminhada, reeducação alimentar) que pudessem me ajudar a enfrentar meu problema de saúde (diabete, hipertensão, depressão).	☐1	☐2	☐3	☐4	☐5
11. Perguntaram sobre meus hábitos de saúde.	☐1	☐2	☐3	☐4	☐5
12. A equipe de saúde levou em conta o que eu penso, no que acredito, meu modo de vida, quando indicou meu tratamento.	☐1	☐2	☐3	☐4	☐5
13. Ajudaram a fazer um tratamento para que eu pudesse seguir no meu dia a dia.	☐1	☐2	☐3	☐4	☐5
14. Ajudaram a planejar como cuidar de minha saúde nos momentos mais difíceis da vida.	☐1	☐2	☐3	☐4	☐5
15. Perguntaram como meu problema de saúde (diabete, hipertensão ou depressão) afeta minha vida.	☐1	☐2	☐3	☐4	☐5
16. A equipe de saúde me procurou, após a consulta, para saber como estava indo meu tratamento.	☐1	☐2	☐3	☐4	☐5
17. Incentivaram a participar de programas comunitários, como grupo de mães, pastoral do idoso, associações, que pudessem me ajudar.	☐1	☐2	☐3	☐4	☐5
18. Orientaram, individualmente ou em grupo, por um nutricionista, psicólogo, fisioterapeuta, farmacêutico, assistente social, equipe de saúde bucal e/ou profissional da educação física, sobre cuidados com minha saúde.	☐1	☐2	☐3	☐4	☐5
19. Informaram que consultas com outros médicos especialistas, como oftalmologistas, cardiologistas, psiquiatras, podem ajudar no meu tratamento.	☐1	☐2	☐3	☐4	☐5
20. Perguntaram como estavam indo as visitas a outros médicos especialistas.	☐1	☐2	☐3	☐4	☐5
