# Supplementary material for: Assessment of the Perception of People Living With HIV Regarding the Quality of Outpatient Care at a Reference Facility in the Federal District, Brazil
Source: Front Pharmacol. 2021 Sep 20;12:740383. doi: 10.3389/fphar.2021.740383 (PMC8522475; doi:10.3389/fphar.2021.740383)
Supplement: Supplementary file 3 [file Table1.DOCX]

Apêndice: Patient Assessment of Chronic Illness Care (PACIC) adaptado para PVHIV

**Iniciais do nome:_________________ Prontuário do HUB:**_________________________

**Data de Nascimento:____/____/________**

**É atendido no ambulatório de infectologia do HUB? Se sim, há quanto tempo?**_________________anos

**Nos últimos 12 meses, quantos atendimentos teve no ambulatório de infectologia? ______. E no Com-Vivência?____. E na FE?_______ E no Laboratório?**________

**Quais serviços você deixou de utilizar e por qual motivo?**___________________________

**___________________________________________________________________________**

**Avaliação do Usuário sobre Cuidado a Condições Crônicas - PACIC**

|  | **Nunca** | **Poucas vezes** | **Algumas vezes** | **Muitas vezes** | **Sempre** |
| --- | --- | --- | --- | --- | --- |
| **1. Perguntaram minha opinião quando definimos o(s) plano(s) para tratamento do HIV.** | _1_ | _2_ | _3_ | _4_ | _5_ |
| **2.** Deram opções de tratamento para que eu pudesse pensar sobre elas. | _1_ | _2_ | _3_ | _4_ | _5_ |
| **3. Perguntaram se tive problemas no uso dos medicamentos ou seus efeitos.** | _1_ | _2_ | _3_ | _4_ | _5_ |
| **4.** Recebi, por escrito, uma lista de coisas que poderia fazer para melhorar minha saúde. | _1_ | _2_ | _3_ | _4_ | _5_ |
| **5. Satisfeito com a organização de meu tratamento.** | _1_ | _2_ | _3_ | _4_ | _5_ |
| **6.** Explicaram que o que eu faço para cuidar de mim mesmo influencia a minha saúde**.** | _1_ | _2_ | _3_ | _4_ | _5_ |
| **7. Perguntaram o que eu pretendo fazer para cuidar da minha saúde.** | _1_ | _2_ | _3_ | _4_ | _5_ |
| **8.**Ajudaram a definir como melhorar minha alimentação, fazer exercícios físicos, controlar estresse, parar de fumar (se tiver hábitos de fumar) e evitar o uso abusivo de álcool e drogas. | _1_ | _2_ | _3_ | _4_ | _5_ |
| **9. Recebi uma cópia, por escrito, do(s) meu(s) plano(s) de tratamento.** | _1_ | _2_ | _3_ | _4_ | _5_ |
| **10.**Fui incentivado a participar de grupos específicos (“centro psicossocial”, rodas de conversa, grupos de apoio) que pudessem me ajudar a enfrentar o HIV**.** | _1_ | _2_ | _3_ | _4_ | _5_ |
| **11. Perguntaram sobre meus hábitos de saúde.** | _1_ | _2_ | _3_ | _4_ | _5_ |
| **12.** A equipe de saúde levou em conta o que eu penso, no que acredito, meu modo de vida, quando indicou meu tratamento. | _1_ | _2_ | _3_ | _4_ | _5_ |
| **13. Me ajudaram a fazer um plano de tratamento para que eu pudesse seguir no meu dia a dia.** | _1_ | _2_ | _3_ | _4_ | _5_ |
| **14.**Ajudaram a planejar como cuidar de minha saúde nos momentos mais difíceis da vida. | _1_ | _2_ | _3_ | _4_ | _5_ |
| **15. Perguntaram como o HIV afeta minha vida.** | _1_ | _2_ | _3_ | _4_ | _5_ |
| **16.**A equipe de saúde entrou em contato comigo depois de uma consulta para saber como estava indo meu tratamento. | _1_ | _2_ | _3_ | _4_ | _5_ |
| **17. Fui incentivado a participar de programas comunitários, em escolas, igrejas ou associações, que pudessem me ajudar.** | _1_ | _2_ | _3_ | _4_ | _5_ |
| **18.** Fui orientado, individualmente ou em grupo, por um nutricionista, psicólogo, farmacêutico, assistente social, equipe de saúde bucal e/ou outro profissional, sobre cuidados com minha saúde. | _1_ | _2_ | _3_ | _4_ | _5_ |
| **19. Informaram que consultas com outros médicos especialistas, como infectologistas, psiquiatras, podem ajudar no meu tratamento.** | _1_ | _2_ | _3_ | _4_ | _5_ |
| **20.** Perguntaram como foram as minhas consultas a outros médicos especialistas. | _1_ | _2_ | _3_ | _4_ | _5_ |
|  | | | | | |
