# Supplementary material for: Assessment of the Perception of People Living With HIV Regarding the Quality of Outpatient Care at a Reference Facility in the Federal District, Brazil
Source: Front Pharmacol. 2021 Sep 20;12:740383. doi: 10.3389/fphar.2021.740383 (PMC8522475; doi:10.3389/fphar.2021.740383)
Supplement: Supplementary file 4 [file DataSheet1.PDF]

Assessment of Care for Chronic Conditions

Staying healthy can be difficult when you have a chronic condition. We would like to learn about the type of help with your condition you get from your health care team. This might include your regular doctor, his or her nurse, or physician's assistant who treats your illness. Your answers will be kept confidential and will not be shared with your physician or clinic.

Over the past 6 months, when I received care for my chronic conditions, I was:

	None of the time	A Little of the Time	Some of the Time	Most of the Time	Always
1. Asked for my ideas when we made a treatment plan.	☐1	☐2	☐3	☐4	☐5
2. Given choices about treatment to think about.	☐1	☐2	☐3	☐4	☐5
3. Asked to talk about any problems with my medicines or their effects.	☐1	☐2	☐3	☐4	☐5
4. Given a written list of things I should do to improve my health.	☐1	☐2	☐3	☐4	☐5
5. Satisfied that my care was well organized.	☐1	☐2	☐3	☐4	☐5
6. Shown how what I did to take care of myself influenced my condition.	☐1	☐2	☐3	☐4	☐5
7. Asked to talk about my goals in caring for my condition.	☐1	☐2	☐3	☐4	☐5
8. Helped to set specific goals to improve my eating or exercise.	☐1	☐2	☐3	☐4	☐5
9. Given a copy of my treatment plan.	☐1	☐2	☐3	☐4	☐5
10. Encouraged to go to a specific group or class to help me cope with my chronic condition.	☐1	☐2	☐3	☐4	☐5
11. Asked questions, either directly or on a survey, about my health habits.	☐1	☐2	☐3	☐4	☐5

GroupHealth.

MacColl Center for Health Care Innovation

Copyright 2004 The MacColl Center for Health Care Innovation, Group Health Cooperative. The Improving Chronic Illness Care program is supported by The Robert Wood Johnson Foundation, with direction and technical assistance provided by Group Health's MacColl Center for Health Care Innovation. For more information go to www.improvingchroniccare.org

<u>Over the past 6 months</u>, when I received care for my chronic conditions, I was:

	None of the time	A Little of the Time	Some of the Time	Most of the Time	Always
12. Sure that my doctor or nurse thought about my values, beliefs, and traditions when they recommended treatments to me.	☐1	☐2	☐3	☐4	☐5
13. Helped to make a treatment plan that I could carry out in my daily life.	☐1	☐2	☐3	☐4	☐5
14. Helped to plan ahead so I could take care of my condition even in hard times.	☐1	☐2	☐3	☐4	☐5
15. Asked how my chronic condition affects my life.	☐1	☐2	☐3	☐4	☐5
16. Contacted after a visit to see how things were going.	☐1	☐2	☐3	☐4	☐5
17. Encouraged to attend programs in the community that could help me.	☐1	☐2	☐3	☐4	☐5
18. Referred to a dietitian, health educator, or counselor.	☐1	☐2	☐3	☐4	☐5
19. Told how my visits with other types of doctors, like an eye doctor or other specialist, helped my treatment.	☐1	☐2	☐3	☐4	☐5
20. Asked how my visits with other doctors were going.	☐1	☐2	☐3	☐4	☐5

GroupHealth

MacColl Center for Health Care Innovation

Copyright 2004 The MacColl Center for Health Care Innovation, Group Health Cooperative. The Improving Chronic Illness Care program is supported by The Robert Wood Johnson Foundation, with direction and technical assistance provided by Group Health's MacColl Center for Health Care Innovation. For more information go to www.improvingchroniccare.org
