# Supplementary material for: Assessment of the Perception of People Living With HIV Regarding the Quality of Outpatient Care at a Reference Facility in the Federal District, Brazil
Source: Front Pharmacol. 2021 Sep 20;12:740383. doi: 10.3389/fphar.2021.740383 (PMC8522475; doi:10.3389/fphar.2021.740383)
Supplement: Supplementary file 5 [file Table3.DOCX]

**Codebook of Variables**

**PLHIV: People living with HIV**

**V1: Age in years**

**V2: Sex**

1- Female; 2- Male

**V3: Schooling**

1- Up to 12 years of study; 2- More than 12 years of study; 3- Unknown

**V4: Race color**

1- White; 2-Non-white; 3- Unknown

**V5: Place of residence**

1- Southeast; 2- East; 3- Center; 4- North; 5- South; 6- Center-South; 7- West; 8-Other; 9- Unknown

**V6: Time since diagnosis in years**

**V7: Regular follow-up**

1-Yes; 2-No

**V8: Viral load**

1- Undetectable; 2- Detectable

**V9: Antiretroviral therapy scheme**

1- Preferential; 2- Alternative

**V10: Adverse reaction to treatment**

1- Yes; 2- No

**V11: Mental health**

1- Unchanged; 2- Changed

**V12: Comorbidity**

1- Yes; 2- No

**V13: Access to other specialties**

1- Obtained; 2- No obtained

**V14: Time under treatment at the hospital in years**

**V15: Number of infectologist visits**

**V16: Number of psychosocial visits**

**V17: Number of laboratory visits**

**V18: Number of visits to the Pharmacy**

**P1: Asked for my opinion when we made a treatment plan for HIV.**

**P2: Given choices about treatment to think about.**

**P3: Asked to talk about any problems with my medicines or their effects.**

**P4: Given a list of things I should do to improve my health.**

**P5: Satisfied that my care was well organized.**

**P6: Shown how what I did to take care of myself influenced my health.**

**P7: Asked to talk about my goals in caring for my health.**

**P8: Helped to set specific goals to improve my eating, engage in exercises, control stress, stop smoking (if applicable) and avoid excess alcohol and drug use.**

**P9: Given a written copy of my treatment plan.**

**P10: Encouraged to go to a specific group (psychosocial support centers, roundtable discussions, support groups) to help me cope with HIV.**

**P11: Asked questions about my health habits.**

**P12: The health team considered what I thought, what I believed, and my lifestyle when they recommended my treatment.**

**P13: Helped to make a treatment plan that I could carry out in my daily life.**

**P14: Helped to plan ahead so I could take care of my health in hard times.**

**P15: Asked how HIV affects my life.**

**P16: The health team contacted me after a visit to see how my treatment was going.**

**P17: Encouraged to attend programs in the community, at schools, churches or associations that could help me.**

**P18: I was advised individually or in a group by a dietician, psychologist, pharmacist, social assistant, oral health team and/or other professional about the care for my health.**

**P19: Told how my visits with other specialists, like infectologists and psychiatrists, can help my treatment.**

**P20: Asked how my visits with other specialists were going.**
